# Martha T. Hunt

**Published:** 1900-03

**Authors:** 


### OBITUARY.

MARTHA T. HUNT, widow of the late Dr. Sanford B. Hunt, died in January at the home of her son, William T. Hunt, in Bernardsville, N. J. Mrs. Hunt was well known in Western New York years ago when she with her husband, Dr. Sanford B. Hunt, resided in Buffalo. She was an aunt to Frank Wood, of Batavia. Dr. Hunt it will be remembered was a former superintendent of the schools, editor of the BUFFALO MEDICAL JOURNAL and the city editor of the *Buffalo Commercial Advertiser*. He served in the Civil War as a surgeon of U. S. Volunteers and after the war wrote a history of the sanitary Commission's work. The burial of Mrs. Hunt took place at Forest Lawn, on Tuesday, January 30, 1900.

